# Distribution and Ecological Drivers of Spotted Fever Group Rickettsia in Asia

**DOI:** 10.1007/s10393-019-01409-3

**Published:** 2019-04-15

**Authors:** Jaruwan Satjanadumrong, Matthew T. Robinson, Tom Hughes, Stuart D. Blacksell

**Affiliations:** 1 Mahidol-Oxford Tropical Medicine Research Unit, Faculty of Tropical Medicine, Mahidol University, 420/6 Rajvithee Road, Bangkok, 10400 Thailand; 2 Lao-Oxford-Mahosot Hospital-Wellcome Trust Research Unit (LOMWRU), Mahosot Hospital, Vientiane, Lao People’s Democratic Republic; 3 Centre for Tropical Medicine and Global Health, Nuffield Department of Clinical Medicine, Churchill Hospital, University of Oxford, Oxford, OX3 7FZ UK; 4 EcoHealth Alliance, 460 West 34th Street, 17th Floor, New York, NY USA

**Keywords:** Spotted fever rickettsia, Rickettsial infection, Land-use change, Diagnosis, Asia

## Abstract

Spotted fever group and related rickettsia (SFGR) are a neglected group of pathogens that belong to the genus *Rickettsia*. SFGR are zoonotic and are transmitted by arthropod vectors, primarily ticks, fleas and mites to accidental hosts. These emerging and re-emerging infections are widely distributed throughout the world. Land-use change and increasing human–wildlife conflict compound the risk of SFGR infection to local people in endemic areas and travelers to these regions. In this article, we discuss the rickettsial organisms causing spotted fever and related diseases, their arthropod vectors in Asia and the impact of land-use change on their spread.

## Introduction

Rickettsioses are infectious diseases caused by obligate intracellular gram-negative bacteria. They belong to the order of Rickettsiales, family *Rickettsiaceae* (Fournier and Raoult 2009) and reside in a wide range of arthropod vectors such as fleas, ticks and mites (Chikeka and Dumler 2015; Merhej et al. 2014). These vectors can transmit pathogens to humans at the bite site, who may or may not subsequently develop disease. Rickettsial diseases have been reported to be the second most common cause of non-malarial febrile illness in the Southeast Asia region after dengue infection (Acestor et al. 2012).

The family *Rickettsiaceae* comprises *Rickettsia* and *Orientia* genera as members and is divided into three major groups; spotted fever group (SFGR), typhus group (TG) and scrub typhus group (STG) (Bhengsri et al. 2016). Rickettsial diseases have worldwide distribution although there are endemic and hyper-endemic areas (Chikeka and Dumler 2015; Luce-Fedrow et al. 2015; Merhej et al. 2014). Typhus group and scrub typhus group are widely diagnosed in Southeast Asia (Aung et al. 2014; Parola et al. 2013; Rodkvamtook et al. 2013). In Asia, typhus group infections are primarily caused by *Rickettsia typhi* which is the etiologic agent of murine typhus (endemic typhus) with a few cases of epidemic or louse-borne typhus caused by *R. prowazekii* reported. Scrub typhus is widespread in Asia–Pacific and northern Australia and is caused by *Orientia tsutsugamushi* along with the related *O. chuto* (Chikeka and Dumler 2015; Izzard et al. 2010).

SFGR consists of at least 30 species that can be found worldwide. Twenty-one species are identified as pathogens: *R. rickettsii*, *R. parkeri*, *R. africae*, *R. massiliae*, *R. philipii*, *R. conorii*, *R. sibirica*, *R. slovaca*, *R. raoultii*, *R. monacensis*, *R. aeschlimannii*, *R. helvetica*, *R. heilongjiangensis*, *R. japonica*, *R. honei*, *R. tamurae*, *Candidatus* Rickettsia kellyi, *R. australis*, *R. mongolotimonae*, *R. felis* and *R. akari.* Nine species are of unknown pathogenicity: *Candidatus* Rickettsia asemboensis, *R. bellii*, *R. montanensis*, *R. peacockii*, *R. rhipicephali*, *R. monteiroi*, *R. gravesii*, *and R. argasii* (Fournier and Raoult 2009; Merhej et al. 2014). There remains some conjecture as to the status of *R. felis* and *R. akari* within SFGR (Chikeka and Dumler 2015). There is an argument that *R. felis* and *R. akari* should be reclassified into a transitional group (Gillespie et al. 2008) on the basis of phylogenetic analysis, however, at this point in time they remain classified as members of the SFGR (Fournier and Raoult 2009; Merhej et al. 2014; Vitorino et al. 2007).

The most well-known rickettsia is *R. rickettsii* which causes Rocky Mountain Spotted Fever (RMSF) and causes human infections predominantly in the USA (Kato et al. 2013). Other species such as *R. australis* and *R. honei* are prevalent in northern Australia (Graves and Stenos 2009). *Rickettsia conorii* is responsible for Mediterranean Spotted Fever (MSF) in several parts of Europe, Africa and Asia (Nanayakkara et al. 2013; Niang et al. 1998; Parola 2004).

The main arthropod vectors of SFGR are ticks, predominantly hard ticks (*Ixodidae*) that bite animals and humans (Luce-Fedrow et al. 2015; Parola et al. 2013). Transmission of the pathogens occurs via salivary products produced during feeding of infected vectors on the wound or mucosal surfaces. Non-validated, incompletely described, or uncultivated SFGR species have also been isolated or detected in soft ticks (*Argasidae*) (Parola et al. 2013); however, the competency to transmit disease is uncertain. Rickettsial infection occurs following infection of the endothelial cell lining of blood vessels (microvascular endothelium infected by *R. conorii* and both microvascular and macrovascular endothelium by *R. rickettsii*) (Colonne et al. 2011; Rydkina et al. 2010).

The purpose of this article is to discuss the distribution of SFGR in Asia, the arthropod vectors and the impact of land-use change on the spread of SFGR disease emergence.

## SFGR Infection and Diagnosis

### Geographical Distribution in Asia

**Figure 1:**
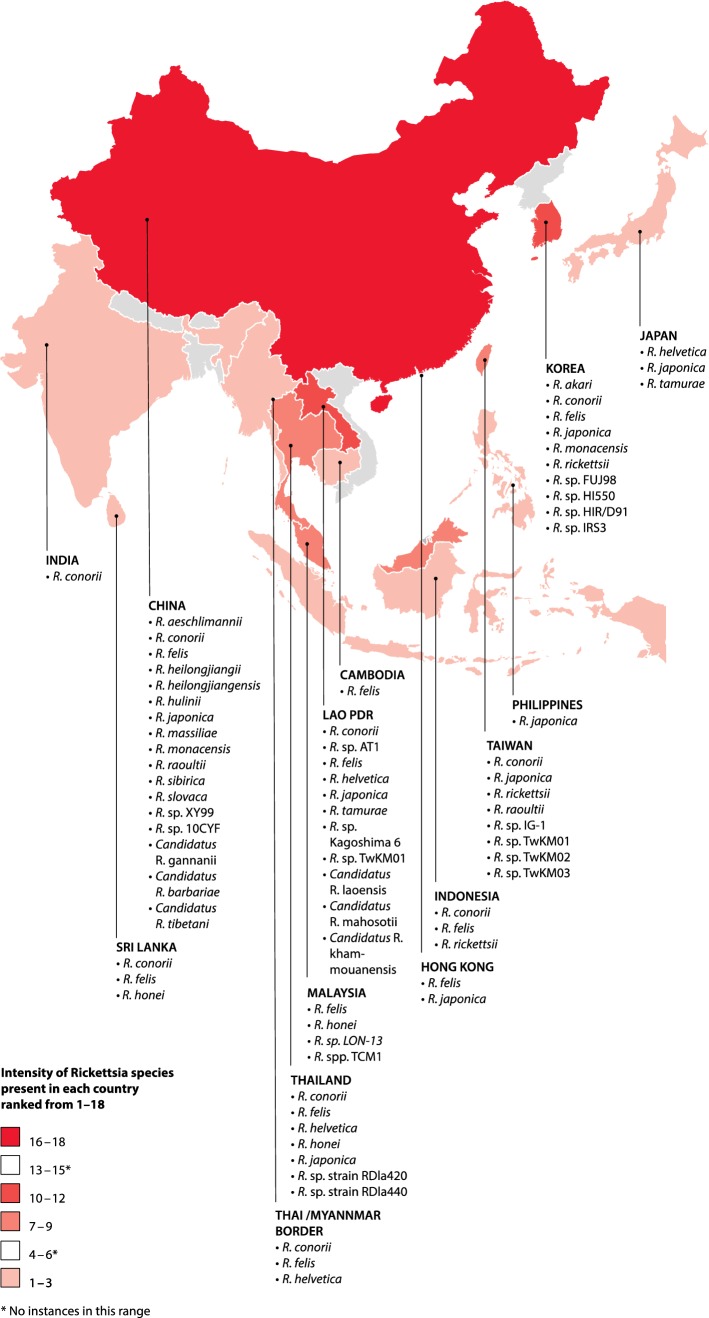
Geographical distribution of reported detections of spotted fever group *Rickettsia* spp. organisms and antibodies in humans, animals and arthropod vectors in Asia.

SFGR are considered to be neglected diseases which are recently emerging or re-emerging infections in several areas of the world, especially in developing countries across Asia (Chikeka and Dumler 2015). The distribution of SFGR based on previous reports is presented in Table 1 and Figure 1.

**Table 1:** Reports of spotted fever group *Rickettsia* spp. organisms and antibodies in humans, animals and arthropod vectors in Asia.

Countries	Rickettsia species	Arthropod vectors	Hosts	References
Cambodia	*R. felis Cal2*	NA	Human, Dogs	Inpankaew et al. (2016)
China	*R. aeschlimannii*	*Rhipicephalus turanicus* (tick)	Sheep	Wei et al. (2015)
	*R. sibirica*	*Rhipicephalus turanicus* (tick)	Sheep	Wei et al. (2015)
	*R. massiliae*	*Rhipicephalus turanicus* (tick)	Sheep	Guo et al. (2016) and Wei et al. (2015)
	*R. conorii*	*Rhipicephalus turanicus* (tick)	Sheep	Guo et al. (2016)
	*R. japonica*	*Haemaphysalis longicornis (tick)*	NA	Sun et al. (2015)
	*R. heilongjiangensis*	*Haemaphysalis longicornis (tick)*	NA	Sun et al. (2015)
	*R. monacensis*	*Amblyomma testudinarium (tick)*, *Ixodes sinensis (tick)*	NA	Sun et al. (2015)
	*R. raoultii*	NA	Human (serum)	Li et al. (2018)
		*Dermacentor silvarum* (tick)	NA	Han et al. (2018)
		*Dermacentor nuttalli* (tick)	NA	Han et al. (2018)
		*Melophagus ovinus* (sheep ked, fly)	NA	Liu et al. (2016)
	*R. slovaca*	*Melophagus ovinus* (sheep ked, fly)	NA	Liu et al. (2016)
	*R. heilongjiangii*	*Dermacentor silvarum* (tick)	NA	Zhang et al. (2000)
	*R. hulinii*	*Haemaphysalis concinna (tick)*	NA	Zhang et al. (2000)
	*R. felis*	*Linognathus setosus* (lice)	NA	Zhang et al. (2014)
		*Ctenocephalides felis felis* (flea)	NA	Zhang et al. (2014)
		*Anopheles sinensis* (mosquito)	NA	Zhang et al. (2014)
		*Culex pipiens pallens* (mosquito)	NA	Zhang et al. (2014)
		*Rhipicephalus sanguineus* (tick)	Dog	Zhang et al. (2014)
		NA	Human, Mice spleen	Zhang et al. (2014)
	*Candidatus R. gannanii*	*Haemaphysalis qinghaiensis* (tick)	NA	Yang et al. (2016)
	*Candidatus R. barbariae*	*Rhipicephalus turanicus* (tick)	Sheep	Guo et al. (2016)
	*Candidatus R. tibetani*	*Haemaphysalis qinghaiensis* (tick),	NA	Han et al. (2018)
		*Dermacentor abaensis* (tick)	NA	Han et al. (2018)
		*Dermacentor silvarum* (tick)	NA	Han et al. (2018)
		*Dermacentor nuttalli* (tick)	NA	Han et al. (2018)
		*Ixodes crenulatus* (tick)	NA	Han et al. (2018)
	*Candidatus R. gannanii Y27*	*Haemaphysalis qinghaiensis* (tick)	NA	Han et al. (2018)
		*Dermacentor abaensis* (tick)	NA	Han et al. (2018)
		*Dermacentor silvarum* (tick)	NA	Han et al. (2018)
	*R. sibirica subspecies sibirica BJ*-*90*	*Dermacentor nuttalli* (tick)	NA	Han et al. (2018)
	*R.* sp. 10CYF	*Dermacentor nuttalli* (tick)	NA	Han et al. (2018)
	*R.* sp. XY99	*Dermacentor nuttalli* (tick)	NA, Human (serum)	Han et al. (2018) and Li et al. (2016)
Hong Kong	*R. felis*	*Ctenocephalides felis* (flea)	Cats	Slapeta et al. (2018)
	*R. japonica*	NA	Human	Ma et al. (2011)
India	*R. conorii* subsp. *indica* strain ITTR (ATCC VR-597)	*Rhipicephalus sanguineus (tick)*	Human	Kalal et al. (2016), Parola et al. (2001) and Sentausa et al. (2012)
Indonesia	*R. felis*	*Xenopsylla cheopis* (flea)	Rat and shrew	Barbara et al. (2010) and Jiang et al. (2006)
	*R. conorii*	NA	Human (blood)	Richards et al. (2003)
	*R. rickettsii*	NA	Human (blood)	Richards et al. (2003)
Japan	*R. japonica*	*Haemaphysalis flava* (tick) *Dermacentor taiwanensis* (tick)	NA NA	Fournier et al. (2002) and Mahara (1997)
		NA	Dog (serum)	Satoh et al. (2001)
	*R. helvetica*	*Ixodes ovatus* (tick)	NA	Fournier et al. (2002) and Mahara (1997)
		*Ixodes monospinosus* (tick)	NA	Fournier et al. (2002) and Ishiguro et al. (2008)
		*Ixodes persulcatus (tick)*	NA	Ishiguro et al. (2008)
		*Ixodes ovatus (tick)*	NA	Ishiguro et al. (2008)
		*Haemaphysalis flava* (tick)	NA	Ishiguro et al. (2008)
		*Haemaphysalis japonica* (tick)	NA	Ishiguro et al. (2008)
		*Amblyomma testudinarium (tick)*	Human	Imaoka et al. (2011)
Korea	*R. conorii*	NA	Human (serum)	Choi et al. (2005)
	*R. felis*	NA	Human (serum)	Choi et al. (2005)
	*R. akari*	NA	Human (serum)	Choi et al. (2005)
	*R. japonica*	NA	Human (serum)	Choi et al. (2005)
		*Haemaphysalis longicornis* (tick)	NA	Lee et al. (2003)
	*R. rickettsii*	*Haemaphysalis longicornis* (tick)	NA	Lee et al. (2003)
	*R.* sp. FUJ98	*Haemaphysalis longicornis* (tick)	NA	Noh et al.(2017)
	*R.* sp. HI550	*Haemaphysalis longicornis* (tick)	NA	Noh et al.(2017)
	*R.* sp. HIR/D91	*Haemaphysalis longicornis* (tick)	NA	Noh et al.(2017)
	*R. monacensis*	*Ixodes nipponensis* (tick)	Mice	Noh et al. (2017) and Lee et al. (2003)
	*R.* sp. IRS3	*Ixodes nipponensis* (tick)	NA	Noh et al. (2017)
Laos	*R. helvetica*	NA	Human (serum)	Phongmany et al. (2006)
	*R. conorii. indica*	NA	Human (serum)	Phongmany et al. (2006)
	*R. felis*	NA	Human (serum)	Phongmany et al. (2006)
		*Ctenocephalides felis felis* (flea)	Dog	Varagnol et al. (2009)
		*Ctenocephalides felis orientis* (flea)	Cat & Dog	Varagnol et al. (2009)
		*Ctenocephalides canis* (flea)	Dog	Varagnol et al. (2009)
	*R. felis URRWXCal2*	NA	Human	Dittrich et al. (2014)
	*R. tamurae*	*Amblyomma testudinarium* (tick)	NA	Taylor et al. (2016)
	*R. japonica*	*Amblyomma testudinarium* (tick)	NA	Taylor et al. (2016)
	*R.* sp. ATT	*Amblyomma testudinarium* (tick)	NA	Taylor et al. (2016)
	*R.* sp. Kagoshima6	*Amblyomma testudinarium* (tick)	NA	Taylor et al. (2016)
	*R.* sp. TwKM01	*Amblyomma testudinarium* (tick)	NA	Taylor et al. (2016)
	*Candidatus* Rickettsia laoensis	*Haemaphysalis* sp. (tick)	NA	Taylor et al. (2016)
	*Candidatus* Rickettsia mahosotii	*Haemaphysalis* sp. (tick), *Amblyomma testudinarium* (tick)	NA	Taylor et al. (2016)
	*Candidatus* Rickettsia khammouanensis	*Haemaphysalis* sp. (tick)	NA	Taylor et al. (2016)
Malaysia	*R. honei* (TT-118)	NA	Human	Tay et al. (2003)
	*R.* sp. TCM1	NA	Rat	Tay et al. (2014)
		*Haemaphysalis bispinosa* (tick)	Sheep	Kho et al. (2017)
	*R. felis*	*Ctenocephalides felis* (flea)	NA	Tay et al. (2014)
		NA	Human	Kho et al. (2017)
	*R.* sp. RF2125	*Ctenocephalides felis* (flea)	NA	Tay et al. (2014) and Kho et al. (2016)
	*R. conorii*	NA	Human	Kho et al. (2017)
	*R.* sp. LON-13	*Haemaphysalis* sp. (tick)	Cat	Kho et al. (2017)
	*Candidatus* Rickettsia asemboensis	NA	Monkeys (blood)	Tay et al. (2015)
Philippines	*R. japonica*	NA	Human	Camer et al. (2003)
Sri Lanka	*R. conorii*	NA	Human	Nanayakkara et al. (2013)
	*R. honei*	NA	Human	Nagalingam et al. (2009)
	*R. felis*	NA	Human	Angelakis et al. (2012)
Thailand	*R. honei*	NA	Rats (serum)	Okabayashi et al. (1996)
		NA	Human (serum)	Edouard et al. (2014)
		*Ixodes granulatus* (tick)	Rat	Kollars et al. (2001)
	*R. japonica*	NA	Rat (serum)	Okabayashi et al. (1996)
	*R. felis*	NA	Human (serum)	Edouard et al. (2014)
	*R. helvetica*	NA	Human (serum)	Parola et al. (2003a)
	*R. conorii*	NA	Human (serum)	Parola et al. (2003a)
	*R.* sp. strain RDla420	*Dermacentor auratus* (tick)	Bear	Parola et al. (2003a)
	*R.* sp. strain RDla440	*Dermacentor* larvae	Wild pig	Parola et al. (2003a)
	*R. conorii*	NA	Human	Parola et al. (2003b) and PicKard et al. (2004)
	*R. felis*	NA	Human	Parola et al. (2003b) and PicKard et al. (2004)
	*R. helvetica*	NA	Human	Parola et al. (2003b) and PicKard et al. (2004)
Taiwan	*R.* sp.TwKM01 (similar to *R. rhipicephali*)	*Rhipicephalus haemaphysaloides* (tick)	NA	Tsui et al. (2007)
	*R.* sp.TwKM02 (similar to *R. australis*)	*Leptotrombidium delicense* (mite)	NA	Tsui et al. (2007)
	*R.* sp.TwKM03 (similar to *R. felis URRWXCal*_*2*_)	*Leptotrombidium delicense* (mite), *Ixodes granulatus* (tick)	NA	Tsui et al. (2007)
	*R. japonica*	*Stivalius aporus* (flea)	Rodent	Kuo et al. (2012)
	*R. rickettsii*	NA	Rodent	Kuo et al. (2015)
	*R. raoultii*	NA	Rodent	Kuo et al. (2015)
	*R.* sp.IG-1	NA	Rodent	Kuo et al. (2015)
	*R. conorii*	*Haemaphysalis ornithophila* (tick)	Bird	Kuo et al. (2017)

*NA* not available.

In Southeast Asia, both animals and humans are infected by SFGR. One of the earliest reports was in 1962 when *Rickettsia* sp. TT-118 was identified from a mixed pool of *Ixodes granulatus* ticks and *Rhipicephalus* spp. larval ticks, collected from rats (*Rattus rattus*) in the north region of Thailand (Chaingmai province) (Jiang et al. 2005; Robertson and Wisseman 1973). *TT*-*118* is a homologous strain of *R. honei*, the pathogen of Flinders Island spotted fever (Stenos et al. 1998), and was reported in Thailand from a patient by sequencing of amplicons from five rickettsial genes to identify the species (Jiang et al. 2005). Human SFGR infection by *R. honei* has been reported in tropical countries such as Thailand and Malaysia (Okabayashi et al. 1996; Tay et al. 2003; Tee et al. 1999). Additionally, *R. thailandii* has been reported, but the pathogenic potential is not defined (Kollars et al. 2001). *R. felis* was first reported in Thailand in 2002 from *Ctenocephalides felis*, the main arthropod vector of the causative agent of cat-flea typhus or flea-borne spotted fever (Edouard et al. 2014). There are several human cases of *R. felis* reported in Thailand, Taiwan (Kuo et al. 2015; Lai et al. 2014; Tsai et al. 2008), Cambodia (Inpankaew et al. 2016), Japan (Perez-Osorio et al. 2008), Malaysia (Tay et al. 2014) and Laos (Dittrich et al. 2014). Besides *R. felis*, other SFGR have also been detected, such as *R. helvetica*, *Rickettsia* sp. *AT1* and *R. conorii* (in particular *R. conorii* subsp*. indica*) in Laos (Dittrich et al. 2014; Phongmany et al. 2006; Varagnol et al. 2009). *Rickettsia japonica*, the pathogen of Japanese spotted fever infection, has been reported in Asian countries including Japan (Fournier et al. 2002), Taiwan (Tsai et al. 2008), Thailand (Okabayashi et al. 1996) and Laos (Taylor et al. 2016). In the Philippines, human antibodies against *R. japonica* have been reported (Camer et al. 2003). *Rickettsia conorii*, the causative pathogen of Mediterranean spotted fever (MSF), has been detected in Indonesia (Richards et al. 2003) and the Thailand–Burma border (Parola et al. 2003b). Three novel species were recently found in Laos which are *Candidatus* Rickettsia laoensis, *Candidatus* Rickettsia mahosotii and *Candidatus* Rickettsia khammouanensis (Taylor et al. 2016). In Sri Lanka, located in South Asia, SFGR *R. felis*, *R. honei*, *R. conorii*, *R. helvetica*, *R. japonica* and *R. slovaca* antibodies have been detected in both humans and canines (Kularatne et al. 2003; Nagalingam et al. 2009; Nanayakkara et al. 2013).

In Northeast/East Asia, SFGR species have been detected throughout China such as *R. felis*, *R. sibirica*, *R. massiliae*, *R. raoultii*, *R. aeschlimannii*, *R. heiongjiangii*, *R. hulinii* and *R. mongolotimonae* (Han et al. 2018; Li et al. 2018; Wei et al. 2015; Yang et al. 2016; Zhang et al. 2000, 2014). In 2015, a novel genotype of SFGR was reported as *Rickettsia* sp. XY99 from ill patients (Li et al. 2016). *Candidatus* Rickettsia gannanii and *Candidatus* Rickettsia barbariae were discovered and suggested to be emerging SFGR species in China (Guo et al. 2016; Yang et al. 2016)*. Rickettsia felis*, *R. japonica* and other SFGR species have been identified in Hong Kong, Japan, Korea and Taiwan (Table 1) (Fournier et al. 2002; Noh et al. 2017; Slapeta et al. 2018; Tsai et al. 2008).

Seroprevalence studies have been used to determine exposure in community or hospital settings to SFGR (Table 2). In Malaysia, prevalence of TT-118 SFGR was 57.3% (Tee et al. 1999) and 12.9% (Tay and Rohani 2002). In South Korea, prevalence against *R. siribica*, *R. conorii* and *R. akari* was 38.6% (Jang et al. 2005). In Sri Lanka, seroprevalence studies of SFGR have found an increase in prevalence from 35 to 66% during 2000–2008 (Kularatne et al. 2013; Premaratna et al. 2008, 2014). Increasing prevalence of SFGR is also noted in central India compared to south and northeast regions (69.3%, 37.1% and 13.8%, respectively) (Kalal et al. 2016; Khan et al. 2016; Rathi et al. 2011). It should be noted that due to extensive cross-reaction within SFGR (Hechemy et al. 1989), it is generally not possible to identify the species level using serological methods unless cross-adsorption techniques are used (La Scola and Raoult 1997), and therefore, results should be interpreted with caution.

**Table 2:** Spotted fever group rickettsia seroprevalence studies in Asia.

Year	Country	Human sample no.	Method	Diagnostic cutoff	SFGR prevalence	Rickettsial species	References
1992–1998, 2001	Philippines	157	IFA	≥ 1:64	1.3%	*R. japonica*	Camer et al.(2003)
1992–1993	South Korea	3362	IFA	1:40	38.6%	*R. siribica* *R. conorii* *R. akari*	Jang et al. (2005)
1994–1999	Malaysia	61,501	IIP	≥ 1:50 (IgM/IgG)	12.9%	TT-118	Tay and Rohani (2002)
1996–1997	Malaysia	300	IIP	≥ 1:50 (IgM/IgG)	57.3%	TT-118	Tee et al. (1999)
2000–2001	Sri Lanka	60	IFA	> 20–5120, > 100 (IgM/IgG)	35%	*R. japonica YH*, TT-118	Kularatne et al. (2003)
2002–2003	Sri Lanka	31	IFA	4-fold rise	28%	SFGR	Premaratna et al. (2008)
2002–2007	Sri Lanka	105	IFA	1:256 (IgG)	40.9%	*R. conorii*	Kularatne et al. (2013)
2008	Sri Lanka	50	IFA	≥ 1:64 (IgG)	66%	SFGR	Premaratna et al. (2014)
2002–2005	Thailand	2225	IFA	≥ 1:128 (IgG) ≥ 1:64 (IgM)	0.8%	SFGR	Bhengsri et al. (2016)
2004–2009	Taiwan	413	IFA	≥ 1:40	11.4% (IgG) 1% (IgM)	*R. felis*, Undetermined SFGR	Lai et al. (2014)
2008–2009	Bangladesh	720	IFA	≥ 1:64	18%	SFGR	Faruque et al. (2017)
2009	Central India	161	ELISA	≥ 1:64 (IgM)	69.3%	SFGR	Rathi et al. (2011)
2010–2012	South India	103	ELISA	Index value > 11 (IgG)	37.1%	SFGR	(Kalal et al. 2016)
2011–2012	Vietnam	908	ELISA	OD ≥ 0.5	1.7%	SFGR	Trung et al. (2017)
2013–2015	Northeast India	1265	ELISA	OD ≥ 0.5	13.8%	SFGR	Khan et al. (2016)

## SFGR Epidemiology

### Vectors

In Asia, *Dermacentor* spp. and *Haemophysalis* spp. (from the family of Ixodidae, or hard ticks) are most frequently associated with rickettsial carriage in Asia, but others also include *Ixodes* spp., *Ambylomma* spp., and *Rhipicephalus* spp. (Table 1). Of the 22 tick species identified to carry SFGR (Table 1), their distribution is more widespread than indicated. *Amblyomma testudinarium*, identified in China, Japan and Laos as a vector of SFGR such as *R. monacensis* and *R. japonica* (Sun et al. 2015; Taylor et al. 2016) (Table 1), is the most widespread of the hard ticks and has also been found in India, Myanmar, Thailand, Malaysia, Indonesia, Philippines, Taiwan, Japan and Korea. *Rhipicephalus sanguineus*, a SFGR vector in China and India known to harbor *R. conorii* and *R. felis* (Zhang et al. 2014; Kalal et al. 2016; Sentausa et al. 2012; Parola et al. 2001), is also known for its worldwide distribution (Gray et al. 2013). The longhorned tick (*Haemaphysalis longicornis*) is found on livestock including cattle, pigs and chickens but also on wildlife including deer, small mammals including rats, and on cats, dogs and humans (Cane 2010). Longhorned ticks have been demonstrated to carry *R. japonica* (Sun et al. 2015; Lee et al. 2003), *R. heilongjiangensis* (Sun et al. 2015), *R. rickettsii* (Lee et al. 2003), *Rickettsia* sp. FUJ98, *Rickettsia* sp. HI550 and *Rickettsia* sp. HIR/D91 (Noh et al. 2017). It should be noted that the detection of rickettsia in the above-mentioned vectors does not imply that they are capable of transmission to hosts; therefore, it is necessary to study vector competence and capacity to better understand the threat associated with each vector.

For the Siphonaptera, *Ct. felis* is the most common rickettsial vector predominantly responsible for transmission of *R. felis* (Edouard et al. 2014; Jiang et al. 2006; Tay et al. 2014; Tsai et al. 2009; Varagnol et al. 2009; Zhang et al. 2014) and also *Rickettsia* sp. RF2125 (Tay et al. 2014; Kho et al. 2016). Again, *Ct. felis* is known to have a worldwide distribution and has been identified throughout Southeast Asia, including China, Hong Kong, Laos and Malaysia (Table 1) (Rust 2017). In addition, the fleas *Ct. canis*, *Xenopsylla cheopsis* (Jiang et al. 2006) and *Vermipsylla alakurt* (mainly distributed in alpine pastoral areas of Northern Asia) have all been demonstrated to be able to harbor rickettsias. Found on sheep, yaks and horses, *V. alakurt* has been demonstrated to be a vector for *Candidatus* R. barbariae (Zhao et al. 2016).

In Asia, SFGR have recently been detected in other orders although the transmission potential remains unclear. *Linognathus setosus*, a louse species which can be found on both domesticated and wild dogs, was found to harbor *R. felis* in China (Zhang et al. 2014). Mite species, although not frequently found to be infected, do remain potential biological vectors, such as *Leptotrombidium delicenses* which was found to harbor organisms related to *R. australis* and *R. felis* in Taiwan (Tsui et al. 2007) and is distributed throughout Southeast Asia (Lv et al. 2018). *Rickettsia akari* is known to be transmitted by *Liponyssoides sanguineus*, the house-mouse mite (Brouqui and Raoult 2006). *Melophagus ovinus*, the sheep ked, a native to Mongolia and North India, and introduced to Japan, was found to harbor rickettsia highly similar to *R. raoultii* and *R. slovaca* in north-western China (Liu et al. 2016).

Rickettsiae can be both transovarially and trans-staidly transmitted in vectors allowing maintenance of the pathogen within the vector population and vectors acting as reservoirs for the organisms (Parola et al. 2013). For instance with ticks, larvae, nymphs and adults are susceptible to infection and also have the capability to transmit rickettsia (Aung et al. 2014). The number of different SFGR species which a vector may harbor, and the potential for human–vector interactions is intrinsically linked to the geographic distribution of the vector and the local environment, and therefore, the distribution of SFGR is likely to be much wider than anticipated. *Rhipicephalus sanguineus* has been demonstrated to increase its human affinity with an increase in environmental temperature (Parola et al. 2008), and therefore, its likely significance as a clinically important vector for rickettsiosis may vary across Asia, despite being a globally distributed tick.

### Hosts

Rats and other rodents are common vertebrate hosts of SFGR infections (He et al. 2003; Okabayashi et al. 1996). In Thailand, 62.2% of rats were found positive for SFGR antibodies (Okabayashi et al. 1996). *Bandicota indica* rats are the most important hosts of SFGR in this area and are infected mainly by *R. honei* (TT-118) (Okabayashi et al. 1996). A bear and wild pigs were reported to be infected by *Rickettsia* sp. strain RDla420 and *Rickettsia* sp. strain RDla440 which are closely related to *R. bellii* and *Rickettsia* sp. RpA4, respectively (Parola et al. 2003a). Domestic animals such as dogs, cats and sheep can serve as important hosts of SFGR infection to humans due to their close relationship and associated activities with humans (Table 1). SFGR infections in rodents and a few cases in birds are also reported in Taiwan (Kuo et al. 2015, 2017). In addition, monkeys can serve as incidental hosts for *Rickettsia* sp. RF2125 and its closely related organisms, *Candidatus* Rickettsia asemboensis and *R. felis* which are detected in monkey blood samples (Tay et al. 2015). Rickettsial pathogens do not only infect local people, often travelers have a higher risk of exposure if visiting SFGR endemic areas, and such documented cases include a Taiwanese patient who contracted African tick bite fever after returning from South Africa (Tsai et al. 2009) and a British visitor to Sabah who almost died from SFGR (Lynn et al. 2018).

## Factors Influencing SFGR Emergence

### Land-Use Change

Land-use change is a significant driver of emerging infectious diseases. Over the last 300 years, the rapidly growing human population has driven land-use change at unprecedented rates (Ramankutty and Foley 1999). Over 60% of emerging infectious diseases in the past 6 decades have originated in animals, with nearly half of these linked to changes in land use, agricultural intensification or changes in food production (Burnside et al. 2012; Jones et al. 2008; Keesing et al. 2010; Patz et al. 2004). Zoonotic pathogens, such as Nipah, SARS, bird flu and Ebola, shared between wild or domestic animals account for the majority of emerging infectious diseases (Taylor et al. 2001), while the majority of pathogens (54.3%) involved in emerging infectious disease events are bacterial or rickettsial (Jones et al. 2008). Increasing contact between people, livestock and wild animals through intensified processes of deforestation, agriculture expansion, land conversion, hunting and urban growth is the driving factor behind this disease emergence (Burnside et al. 2012; Jones et al. 2008; Keesing et al. 2010; Patz et al. 2004).

Asia is the largest continent comprising up to 30% of the world’s land area, and with 60% of the world’s population, it is also the most populated. The estimated population for Asia in 2018 according to UN estimates is 4.5 billion people (Worldometers 2018). Asia has the highest growth rates in the world with its population almost quadrupling during the twentieth century. This rapidly growing population increases pressure for agriculture expansion, land conversion, hunting and urban growth. Currently 48.6% of Asia’s population lives in urban areas, and by 2050 this will have increased to 63% of the population (Worldometers 2018). As populations continue to grow and the demands for urban living continue to increase, more and more previously pristine environments will be encroached on further driving disease emergence (Foley et al. 2005).

For much of Asia, these pristine environments are forests. Over the past 300 years, global deforestation has resulted in a net loss of between 7 and 11 million km^2^ of forest—an area the size of the continental USA (Foley et al. 2005; Myers and Patz 2009). According to the 2005 FAO Global Forest Resources Assessment, approximately 13 million hectares of forest are being lost globally to deforestation annually (FAO 2005). While in Asia as a whole, the situation has improved from a net loss in the 1990s to a net gain of forests in 2000–2005, in South and Southeast Asia; however, deforestation continues to increase (FAO 2005). This deforestation caused by agricultural and infrastructure expansion results in the remaining forest patches becoming increasingly fragmented. Currently, approximately 70% of the world’s forests lie within 1 km of a forest edge (Haddad et al. 2015; Watson et al. 2018). This makes the remaining forest increasingly accessible to human populations and, in turn, makes human and livestock populations increasingly accessible to hosts and vectors for SFGR that live in the forest.

### Increased Access to Forest Areas

As road and other transport networks are improved and expanded, it becomes easier for larger numbers of people to move into or visit previously remote areas, increasing opportunities for contact with vectors and hosts and the potential for SFGR exposure. Pristine and degraded forests are cleared to generate income and to make way for agricultural expansion, extractive industries, expanding transport networks and growing urban areas. As forest fragmentation increases, so does the network of legal and illegal roads around and through them. Initially for access for legal and illegal logging, these roads also provide access to people looking for new areas for agricultural, infrastructure and urban expansion in these previously inaccessible forested regions. As agricultural activities develop in a particular location and governments look to encourage more people to an area, the road networks that serve them begin to improve. As has been seen in Thailand, Cambodia and Sumatra, the growing and improved road network results in increased deforestation, with most logging occurring near roads due to increased accessibility (Clements et al. 2014; Cropper et al. 2001; Miyamoto 2006) further increasing forest fragmentation. A study in Peninsular Malaysia found more than 90% of snares and poaching camps were located near paved roads (Clements et al. 2014) confirming that as forest fragmentation and road expansion increase, people (including hunters) find it easier to access more forest.

### Introduced Vectors

These new roads in forested areas not only increase human access but also migration (Laurance et al. 2002) bringing more people into areas where they can potentially have contact with the hosts and forest vectors for SFGR. This movement of people can also bring new hosts and vectors such as domestic dogs and their ticks, including the SFGR-vector *R. sanguineus* (Scinachi et al. 2017). Recent studies have demonstrated that *Rh. sanguineus* exposed to high temperatures feed on humans more rapidly (Parola et al. 2008); therefore, the SFGR risk could increase in rural areas where deforestation results in higher temperatures. The widely distributed *A. testudinarium* is found predominantly in tropical wooded environments (Levin 2018). The increase in forest fragmentation making larger areas of forest accessible, and the corresponding increase in inhabitants farming and living near to the forest edge will increase the potential for contact with such vectors resulting in SFGR spillover.

### The Human, Animal, Vector Nexus

Ticks thrive in the moist and humid environments of the tropics, where they are most commonly found in rural areas; such as forests, grasslands, fields and plantations. Ticks feed on a variety of hosts (Parola et al. 2013) and the tropical areas of South and Southeast Asia, with its high wildlife biodiversity, provide a vast range of hosts for the arthropod species that act as vectors for SFGR. Various studies have demonstrated that people working in rural areas, especially those working in forests, or people involved in deforestation or forest fragmentation and people working in agriculture have a higher risk of being exposed to ticks and potentially SFGR (Quintero et al. 2017; Finch et al. 2014; Tee et al. 1999; Tay et al. 2000).

While the increased funding and the improvement in and adoption of new molecular tools in the last 2 decades have led to an increase in the discovery of novel rickettsia species, and the increase in diagnoses for tick-borne rickettsial diseases, the impact of land-use change across Asia resulting in more people being exposed to SFGR vectors and hosts must also be recognized (Premaratna 2016). These human activities displace wild animals, change vector breeding patterns and force heightened interaction between humans, wildlife and livestock, increasing the chances for the transmission of new diseases, by creating increased opportunities for viruses and bacteria, including SFGR, to jump from wild animal hosts into livestock and domestic animals or directly into people. As SFGR have been demonstrated to be more prevalent in rural areas (Tay et al. 2000, 2003; Tee et al. 1999), rapid economic development and population growth throughout Asia, increasing deforestation and the need for more agricultural land, will bring increasing numbers of people into contact with SFGR vectors and hosts, as human populations move into previously undisturbed areas. While SFGR infections are more common in rural areas (Vallee et al. 2010), people living in urban environment will also be more exposed to SFGR, as rodents and other hosts such as non-human primates adapt to these urban environments (Scientific Committee on Vector-borne Diseases 2013; Tay et al. 2015) and economic development allows more people from urban populations to engage in activities in rural areas such as jungle trekking and other forms of ecotourism, further exposing them to SFGR (Scientific Committee on Vector-borne Diseases 2013; Tay et al. 2003). The potential impact of land-use change on the spread of SFGR greatly increases the urgency for the development of comprehensive detection and response capacities, particularly in “hot spot” areas such as South and Southeast Asia.

Recognizing the link between land-use change and the spread of SFGR also presents an opportunity for low-cost healthcare interventions. Understanding that people working outside in rural areas, especially those working in the forest, involved in deforestation or forest fragmentation and people working in agriculture, are at a higher risk of exposure to vectors and hosts for SFGR allows for more targeted healthcare interventions. Healthcare practitioners working in rural communities need to be reminded to consider suspected rickettsioses including SFGR as one of the potential diagnoses for these high-risk patients presenting with acute febrile illness. There is a need to emphasize the screening of rickettsioses to medical personnel throughout Asia and to encourage the use of appropriate antibiotics as early treatment for nonspecific febrile illnesses in this region (Lynn et al. 2018). Members of these high-risk groups can be taught to recognize their unique risk and to be more vigilant about protecting themselves from ticks and other vectors and seeking medical treatment when they show symptoms of SFGR or other tick, mite or louse-related illnesses. Similarly, those living in urban areas can be reminded of the habitats that ticks, mites or lice and their hosts prefer and the need to be mindful when in these environments, whether in an urban or rural setting, and to seek medical treatment if experiencing symptoms of SFGR or other tick, mite or louse-related illness after being in these areas. Finally, reducing deforestation, forest fragmentation and land-use change has the potential to reduce disease emergence including SFGR (Quintero et al. 2017). This can be achieved by utilizing science-based, land-use planning tools, for more sustainable land management. Better land-use planning can reduce the scale of land-use change by ensuring that areas selected for land-use change are suitable for and have the highest possible value for its new use.

## Conclusions

Generally, SFGR infections are significantly neglected and under-recognized in Asia while causing a significant burden of disease. SFGR are not widely studied because of the limitation of diagnostic techniques, but the distribution of this rickettsial group appears to be spreading wider and more cases have been increasingly reported throughout many parts of the world. As the pressure for land-use change continues, the spread and number of SFGR cases are likely to increase due to increased interaction between humans, hosts and vectors. Additionally, SFGR may be one of the main neglected diseases that would place increased financial burden on medical systems, especially in developing countries. Both the development of new strategies, including healthcare interventions targeted at high-risk groups, more informed land-use planning, and the improvement of existing techniques will assist diagnostic capabilities and lead to the appropriate antibiotic treatments for patients which can save and protect people’s lives from severe rickettsial infection. Since rickettsial infections have become more globally documented, the growing importance of rickettsia agents and its vector populations have also been increasingly studied and the accumulated information of rickettsial prevalence on human and animal hosts is useful to predict the risk of infection so that we can further identify its pathogenicity on humans. This information allows for control and prevention strategies to be identified, prioritized and implemented.
